# Redistribution of Ionotropic Glutamate Receptors Detected by Laser Microdissection of the Rat Dentate Gyrus 48 h following LTP Induction *In Vivo*

**DOI:** 10.1371/journal.pone.0092972

**Published:** 2014-03-25

**Authors:** Jeremy T. T. Kennard, Diane Guévremont, Sara E. Mason-Parker, Wickliffe C. Abraham, Joanna M. Williams

**Affiliations:** 1 Brain Health Research Centre, University of Otago, Dunedin, New Zealand; 2 Department of Anatomy, Otago School of Medical Sciences, Dunedin, New Zealand; 3 Department of Psychology, University of Otago, Dunedin, New Zealand; SUNY Downstate Medical Center, United States of America

## Abstract

The persistence and input specificity of long-term potentiation (LTP) make it attractive as a mechanism of information storage. In its initial phase, both *in vivo* and *in vitro* studies have shown that LTP is associated with increased membrane localization of AMPA receptor subunits, but the molecular basis of LTP maintenance over the long-term is still unclear. We have previously shown that expression of AMPA and NMDA receptor subunits is elevated in whole homogenates prepared from dentate gyrus 48 h after LTP induction *in vivo*. In the present study, we utilized laser microdissection (LMD) techniques to determine whether AMPA and NMDA receptor upregulation occurs specifically in the stimulated regions of the dentate gyrus dendritic arbor. Receptor proteins GluN1, GluA1 and GluA2, as well as postsynaptic density protein of 95 kDa and tubulin were detected by Western blot analysis in microdissected samples. Gradients of expression were observed for GluN1 and GluA2, decreasing from the inner to the outer zones of the molecular layer, and were independent of LTP. When induced at medial perforant path synapses, LTP was associated with an apparent specific redistribution of GluA1 and GluN1 to the middle molecular layer that contains these synapses. These data indicate that glutamate receptor proteins are delivered specifically to dendritic regions possessing LTP-expressing synapses, and that these changes are preserved for at least 48 h.

## Introduction

Long-term potentiation (LTP) is a persistent increase in the efficiency of synaptic transmission following high-frequency or patterned stimulation of input pathways. LTP persists for days or months in the rat dentate gyrus *in vivo* [1], [2]and it is this persistence, in addition to other properties such as input specificity and associativity, that supports LTP as a candidate mechanism for information storage at mammalian synapses [3], [4]. The mechanisms that mediate the induction and early expression of LTP are increasingly well-described, including the activation, modification and recruitment of postsynaptic ionotropic glutamate receptors. As it is the long-lasting nature of LTP that gives its relevance to long-term information storage, it is important to understand the late expression and maintenance of LTP; yet the molecular events that underlie persistent LTP are poorly understood.

It has been shown using *in vitro* models that one mechanism underlying the early phase of LTP is the rapid membrane insertion of α-amino-3-hydroxy-5-methylisoxazole propionate receptors (AMPARs) consisting of GluA1 and GluA2-type subunits [5], [6], [7], although this has not been observed in all systems [8]. Correspondingly, increased numbers of immunogold-labelled AMPARs are present at perforant path synapses 75 min following induction of LTP in the dentate gyrus of anaesthetized rats [9], while increased levels of the GluA1 and GluA2 AMPAR subunits occur 20 min after the induction of LTP in the dentate gyrus of awake animals [10]. In these latter experiments, we observed protein synthesis-independent increases in GluA1 and GluA2 expression in synapse-enriched biochemical fractions (synaptoneurosomes) and surface membranes, while only GluA1 was elevated in isolated postsynaptic densities (PSDs). These data are strongly suggestive of an important role for homomeric GluA1 receptors in the early period following LTP induction.

Late-phase LTP (LTP2 and LTP3) *in vivo* requires *de novo* protein synthesis [11],[12],[13] and we have shown that 48 h post-induction, LTP is associated with increased overall expression of GluA1 and GluA2 protein in the dentate gyrus [14], but no change in their expression in synaptoneurosome surface membrane or PSD fractions. This is in contrast to our observations at 20 min post-LTP and raises the question as to why the pattern of receptor expression changes across time. As our analyses were carried out using whole dentate gyri, one explanation is that heterosynaptic long-term depression (LTD) occurring at non-stimulated synapses obscures changes at LTP-expressing synapses [15], [16], [17]. This hypothesis is supported by reports that homosynaptic LTD is mediated by removal of AMPARs from synapses ([18], [19] but see [20], [21]).

To determine whether glutamate receptor expression changes specifically in the region of the LTP-stimulated synapses, we first induced LTP at granule cell synapses of medial perforant path afferents, and then used laser microdissection (LMD) to separately remove the termination zones of the medial and lateral perforant paths in the middle and outer thirds of the granule cell dendritic arbors, respectively. LMD, in which an ultraviolet laser is used to dissect areas of interest from tissue sections, has been used extensively for the analysis of nucleic acids [22], [23]. In contrast, because large amounts of dissected material are required for the analysis of protein expression, only two protein-based studies have been carried out previously in the rat dentate gyrus, where expression of the highly abundant components of the actin cytoskeleton [24] and caspase enzyme expression in granule cells were investigated [25]. Here, we show that AMPAR and *N*-methyl-D-aspartate receptor (NMDAR) proteins can be detected using Western blot analysis in microdissected tissue, and that layer-specific changes in AMPAR and NMDAR protein expression are observed 48 h after LTP induction.

## Materials and Methods

### Surgery

All experiments were conducted on perforant path-dentate gyrus synapses in adult male Sprague-Dawley rats (400–550 g, ∼4 months old at the time of surgery), using surgical protocols approved by the University of Otago Animal Ethics Committee and in accord with New Zealand animal welfare legislation. After ketamine (75 mg/kg, i.p.) and domitor (0.5 mg/kg, i.p.) anaesthesia, surgery and chronic placement of electrodes were carried out as previously described [26]. Briefly, Teflon-coated 75 μm monopolar stainless steel stimulating electrodes were implanted in the angular bundle to separately activate the medial and lateral perforant path fibres (4 mm and 5 mm lateral to lambda, respectively), and recording electrodes implanted in the hilus of the dentate gyrus (3.8 mm posterior and 2.5 mm lateral to bregma).

### Electrophysiology

Animals were allowed 2 weeks to recover from surgery, and the quality of evoked field excitatory postsynaptic potentials (fEPSPs) was assessed; recordings were considered usable if the medial path fEPSP slope ≥ 3.5 mV/ms when evoking a 2–4 mV population spike and lateral path fEPSP amplitude ≥ 4 mV, both in response to stimulus currents ≤500 μA. Baseline recordings at 0.1–0.067 Hz were made in response to 150 μs duration pulses alternating between the medial and lateral paths. The baseline stimulus strengths used were sufficient to elicit a 2–4 mV population spike in the medial path, and a 4–5 mV fEPSP in the lateral path.

Experimental animals underwent bilateral surgery and a unilateral tetanization paradigm, consisting of 50 trains of 10 pulses at baseline stimulation current intensity, 250 μs pulse duration at 400 Hz, presented in five-train bursts at 1 Hz with an inter-burst interval of 1 min. This paradigm has been successfully used to induce LTP in the medial path and heterosynaptic LTD in the lateral path [26], [27]. For 20 min following the last train, and at 24 h and 48 h post-tetanization, responses were recorded and the mean of the last 20 responses determined. Criteria for LTP in the medial path were ≥ 15% increase in fEPSP, and ≥ 50% increase in population spike at the 48 h time-point, while the criterion for LTD in the lateral path was a depression of ≥ 15% in fEPSP at the 48 h time-point. Animals were anaesthetised with halothane immediately following 48 h recordings, and decapitated; brains were removed and transferred to dry ice-chilled isopentane. Once frozen, brains were removed from isopentane and stored at −80°C prior to cryosectioning.

### Cryosectioning and Laser Microdissection

Frozen brains were thawed to −15°C in a cryostat chamber (CM3050 S, Leica Microsystems, Wetzler, Germany). Coronal sections (35 μm) were cut from posterior to anterior between approximately bregma −4.6 mm and bregma −2.6 mm and thaw-mounted onto polyethylene napthalate membrane-coated LMD slides (Leica Microsystems). Slides were air-dried for 45 min under a fume hood at room temperature. Sections were then stained for 2 min in thionin (0.05% w/v thionin, sodium acetate 0.15 M, acetic acid 0.1 M) and then washed (2 × 1 min) in distilled water. Thionin-stained sections were stored at −20°C prior to LMD.

Sections were air-dried for 45 min prior to microdissection as this was found to improve the effectiveness of laser cutting through the tissue. The inner, middle and outer thirds of the dentate gyrus molecular layer and the granule cell layer were dissected from cryosections using an ASLMD laser microdissecting microscope (Leica Microsystems). Processing of a set of sections from an individual animal encompassed three to four sessions of LMD; after each session, lysis buffer (1% sodium dodecyl sulfate (SDS), Tris 62.5 mM, dithiothreitol 100 mM) was added to the caps of 0.5 mL microcentrifuge tubes in which LMD tissue was collected. A short centrifugation step collected the tissue and buffer at the bottom of tubes, after which the tissue was bath-sonicated for 5 min, and incubated at 99°C for 5 min. Sonicated samples were stored at −80°C. A number of LMD sessions were required to complete processing of an individual subject, and samples from each session for individual subjects were pooled to give one sample per hemisphere per animal (30 sections) for each dissected region. Material collected was sufficient for three SDS-polyacrylamide electrophoresis (SDS-PAGE) experiments per animal. Samples were either stored at −80°C after pooling prior to SDS-PAGE or aliquots were taken for immediate SDS-PAGE.

### Western Blot Analysis

LMD samples were separated by SDS-PAGE (9%) and transferred to nitrocellulose membrane (Schleicher and Schuell). Membranes were probed either with an antibody recognising GluA1 (Upstate, 05–855), GluA2 (Zymed, 32-0300), GluN1 (Zymed, 32-0500), PSD-95 (BD Transduction, 610496), and α-tubulin (Abcam, AB4074). Antibody binding was detected using Supersignal Pico West (Pierce) and immunoreactivity quantified by densitometry [10], [28], [29]. GluA1, GluA2 and GluN1 were reliably detected in all fractions except the granule cell layer. Tubulin was detected in all fractions.

### Statistical Analysis

#### Within hemisphere comparisons

Data for the middle molecular layer (MML) and outer molecular layer (OML) in an individual hemisphere were determined relative to the corresponding inner molecular layer (IML) (Fig. 3B, 4B, 5B). In some analyses, these data were normalised to tubulin. As we observed a gradient in tubulin expression across the molecular layer, analyses were also carried out without tubulin normalisation. These data sets, which encompassed all animals for each antibody, were subjected to two-way mixed-model analysis of variance, followed by Bonferroni test for multiple comparisons when appropriate.

#### Across hemisphere comparisons

Data for individual subregions microdissected from the tetanised hemisphere were normalised to tubulin and expressed relative to the values determined for the corresponding subregion in the matched contralateral control hemisphere (e.g. tetanised MML versus control MML; Fig. 3C, 4C, 5C). Data were subjected to two-tailed Student's *t*-tests.

In all instances, data were generated by within-gel comparison of samples and are reported as mean ± SEM with a probability level of ≤ 0.05 accepted as statistically significant.

## Results

### Synaptic proteins can be detected in laser microdissected dentate gyrus tissue

**Figure 1:**
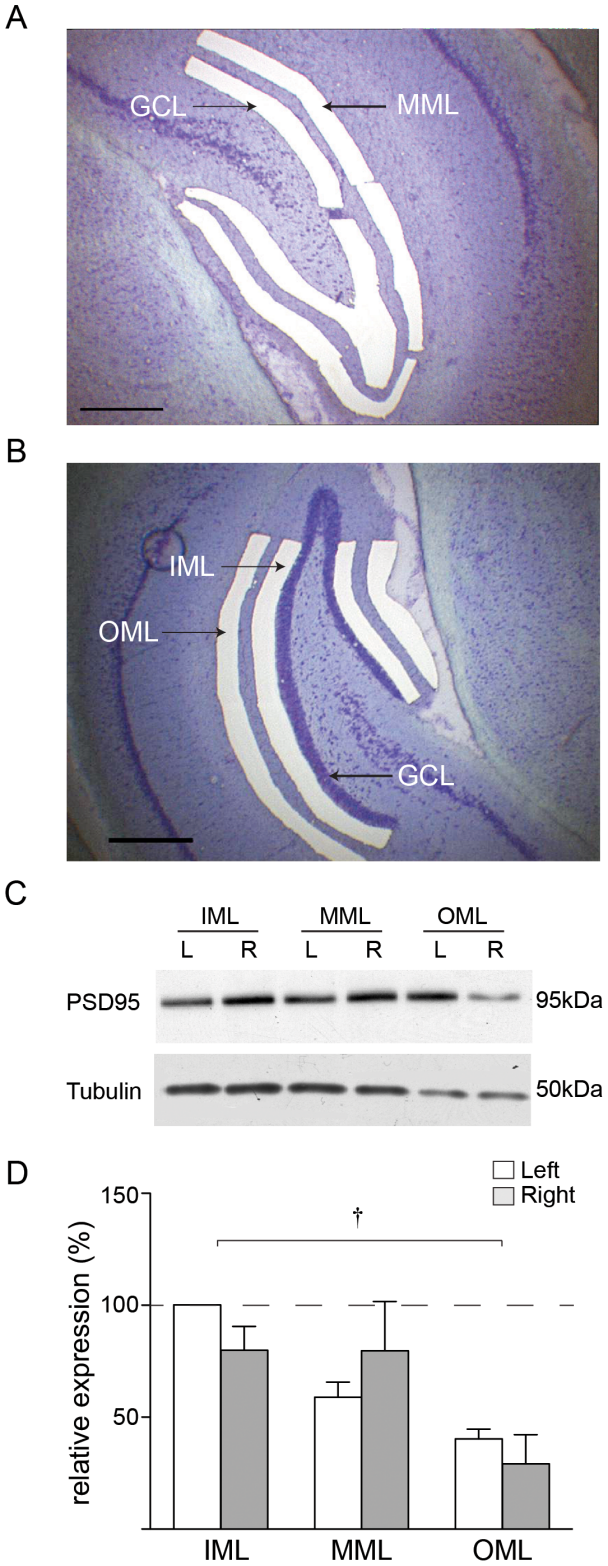
Detection of synaptic proteins in laser microdissected tissue. (a) Laser microdissection of middle molecular layer (MML) and granule cell layer (GCL) of dentate gyrus from a thionin-stained 35 μm coronal section of rat brain. (b) Laser microdissection of outer (OML) and inner (IML) molecular layers. (c) PSD-95 and tubulin expression in naïve rats: representative Western blot showing PSD-95 and tubulin expression in laser microdissected tissue from one animal. (d) Mean expression of tubulin (n = 4 animals) determined as a percentage of the left IML; † p<0.01 ANOVA.

**Figure 2:**
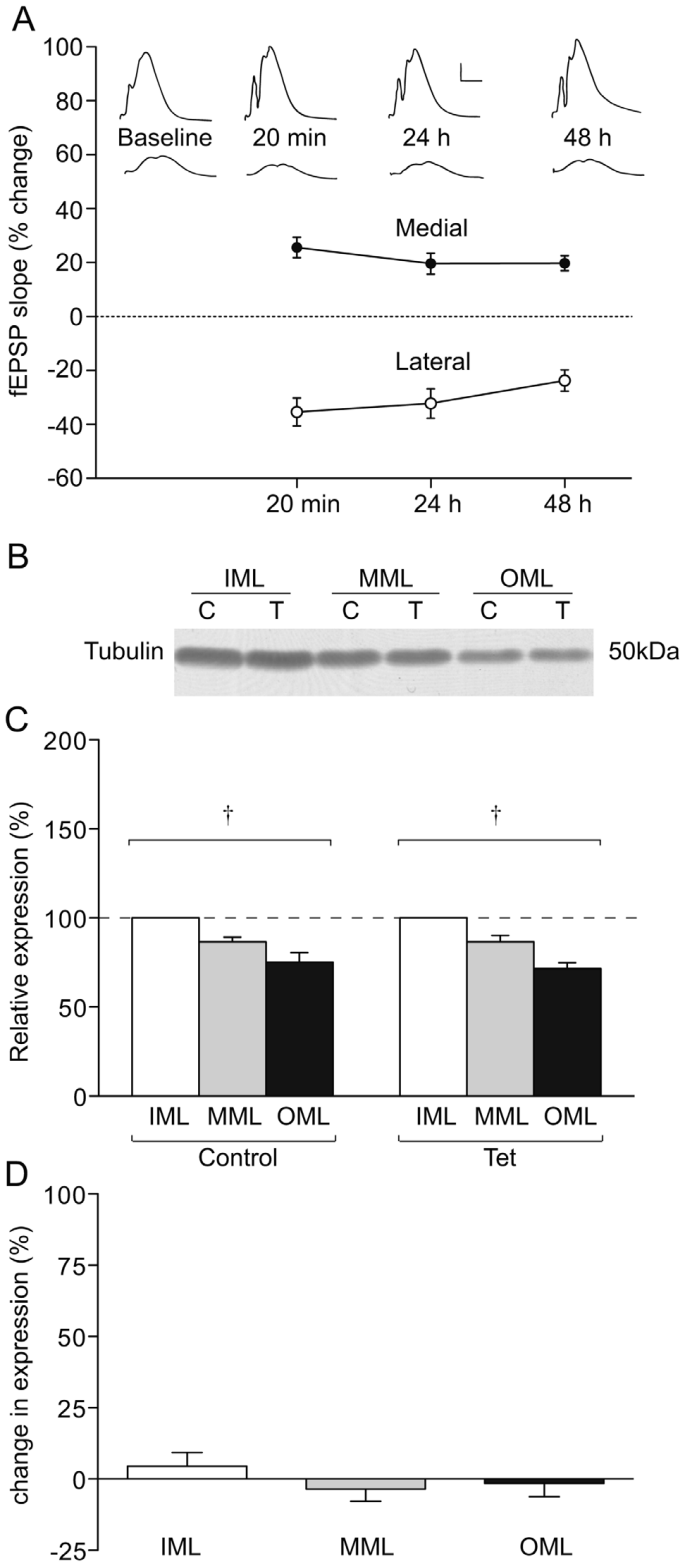
Tubulin expression in LTP-stimulated rats. (a) LTP of fEPSPs in the medial perforant path synapses (MML) and heterosynaptic LTD in the lateral perforant path synapses (OML), n = 8. (b) Representative Western blot showing tubulin expression in laser microdissected tissue from one LTP-stimulated animal. (c) Mean percentage difference in expression in molecular layer zones of the LTP-stimulated hemisphere compared to the naïve hemisphere. (d) Mean relative expression of tubulin in the molecular layer zones from LTP-stimulated and naïve hemispheres, determined as a proportion of the ipsilateral inner molecular layer; † p<0.01 ANOVA.

**Figure 3:**
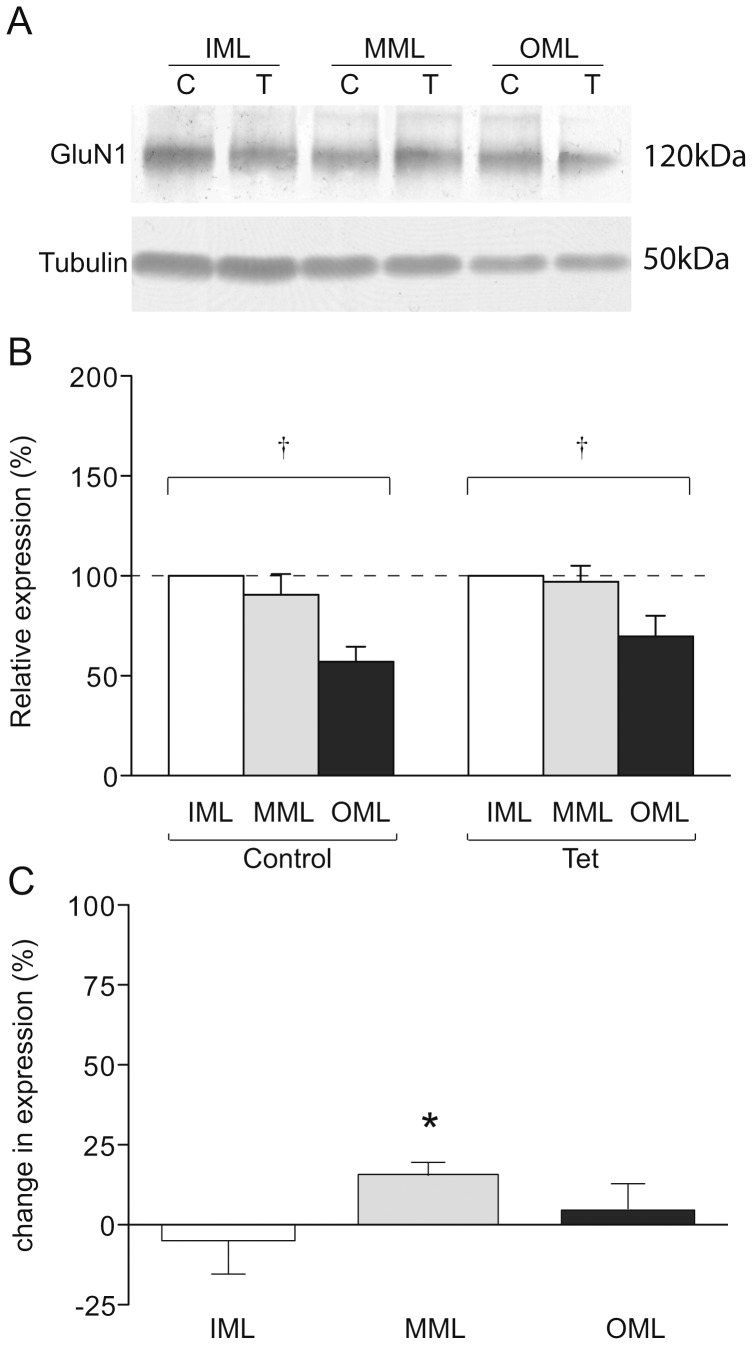
GluN1 expression in LTP-stimulated rats. (a) Representative Western blot showing GluN1 (upper) and tubulin (lower) expression in laser microdissected tissue from one LTP-stimulated animal. (b) Mean relative expression of GluN1 in the molecular layer zones from LTP-stimulated and naïve hemispheres, determined as a proportion of the ipsilateral inner molecular layer. (c) Mean percentage difference in tubulin-normalised expression of GluN1 in molecular layer zones of the LTP-stimulated hemisphere compared to the naïve hemisphere; * p<0.05; † p<0.01 ANOVA.

**Figure 4:**
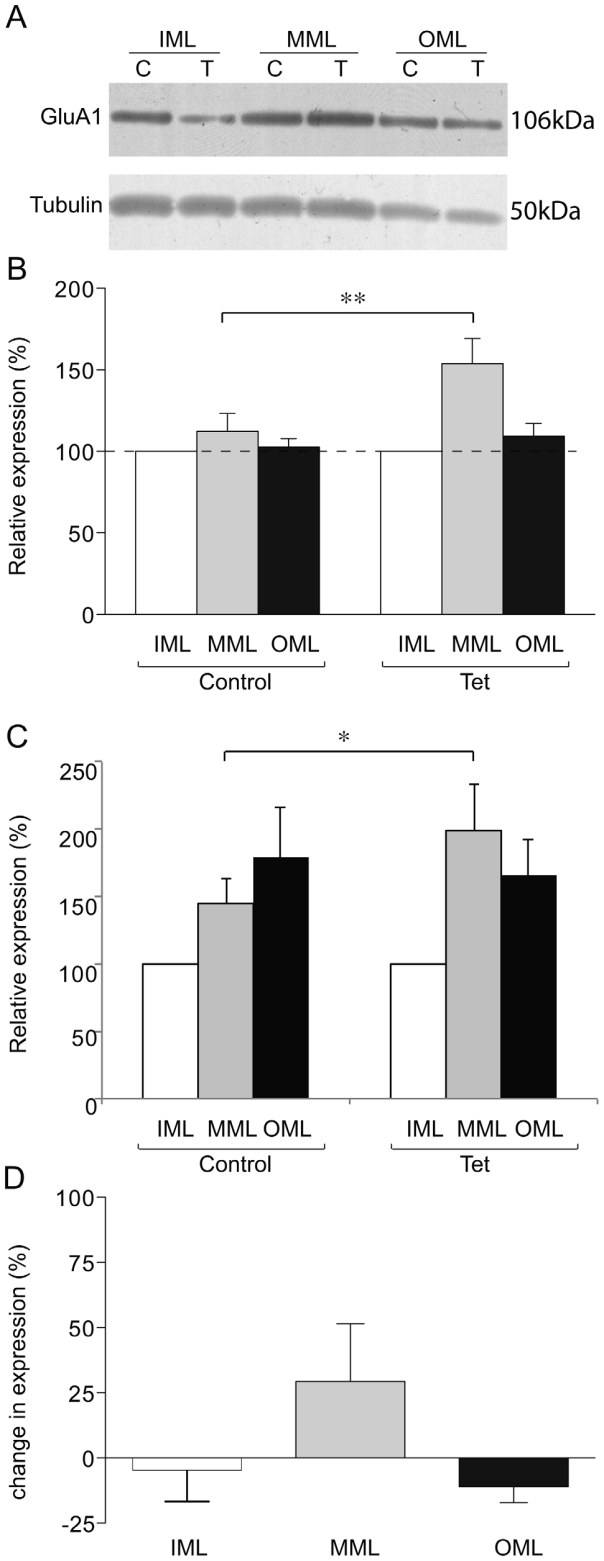
GluA1 expression in LTP-stimulated rats. (a) Representative Western blot showing GluA1 (upper) and tubulin (lower) expression in laser microdissected tissue from one LTP-stimulated animal. (b) Mean relative expression of GluA1 in the molecular layer zones from LTP-stimulated and naïve hemispheres, determined as a proportion of the ipsilateral inner molecular layer. (c) Mean relative expression of GluA1 in the molecular layer zones from LTP-stimulated and naïve hemispheres, using tubulin as a loading control and determined as a proportion of the ipsilateral inner molecular layer. (d) Mean percentage difference in tubulin-normalised expression of GluA1 in molecular layer zones of the LTP-stimulated hemisphere compared to the naïve hemisphere; * p<0.05 (single tailed); ** p<0.01 (two tailed).

**Figure 5:**
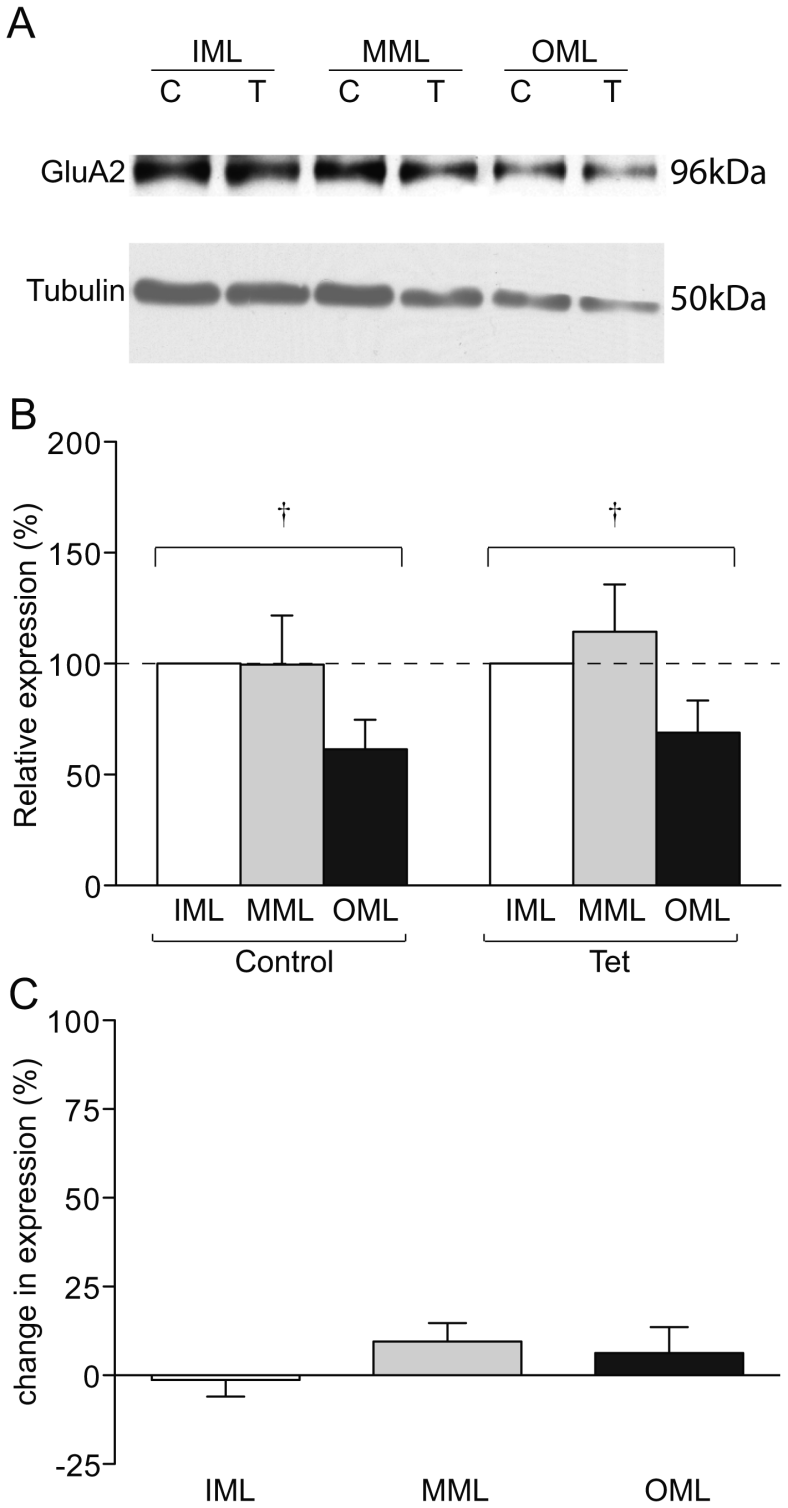
GluA2 expression in LTP-stimulated rats. (a) Representative Western blot showing GluA2 (upper) and tubulin (lower) expression in laser microdissected tissue from one LTP-stimulated animal. (b) Mean relative expression of GluA2 in the molecular layer zones from LTP-stimulated and naïve hemispheres, determined as a proportion of the ipsilateral inner molecular layer. (c) Mean percentage difference in tubulin-normalised expression of GluA2 in molecular layer zones of the LTP-stimulated hemisphere compared to the naïve hemisphere; † p<0.01 ANOVA.

In order to establish whether synaptic proteins can be detected in LMD tissue using Western blot analysis, frozen cryosections (35 μm thickness) were cut from a naïve rat brain and stained with thionin. Tissue from four subregions of the dentate gyrus was microdissected from 10 alternate sections, namely the middle molecular layer (MML) and granule cell layer (GCL) (Fig. 1A), and the outer and inner molecular layer (OML and IML, respectively) from the corresponding alternate sections (Fig. 1B), giving 20 sections in all. Tissue fragments were collected in dry microcentrifuge tubes and solubilised prior to SDS-PAGE and Western blot analysis. Synaptic proteins GluA1, GluA2, GluN1 (aka NR1) (Fig. 3, 4, 5), postsynaptic density protein of 95 kDa (PSD-95; Fig. 1C) and the cytoskeleton protein tubulin (Fig. 1,2) were all detected in microdissected synaptic samples, while only tubulin was reliably detected in the GCL (data not shown). In subsequent experiments, microdissected tissue from 30 sections was pooled for each individual animal, being sufficient for three SDS-PAGE experiments.

### Tubulin expression is greater in proximal versus distal dendritic zones

While in previous studies we used protein quantification assays to normalise the amount of sample compared by SDS-PAGE, the low protein yields from microdissected tissue made this impractical. Tubulin, as a component protein of dendritic and axonal microtubules, was considered a useful candidate as a loading control. In naïve rats, tubulin levels in tissue layers microdissected from the left and right dentate gyrus were assessed by Western blot, and normalised arbitrarily to the expression level in the left IML (Fig. 1C,D). No differences were detected between hemispheres in any layer (n = 4; two-way ANOVA; Fig. 1D) but a layer specific effect was found using a two-way mixed-model ANOVA (p<0.01), where the levels of tubulin detected in the OML were significantly lower than the IML and MML (one-way ANOVA p< 0.05).

### Tubulin expression is not altered 48 h after LTP induction

Unilateral high-frequency stimulation (HFS) of the medial perforant path [27] induced a robust potentiation of the fEPSP measured 20 min post-induction at MML synapses (26 ± 4% over baseline, p<0.01) that persisted to 48 h (20 ± 4%, p<0.01, n = 8; Fig. 2A). Heterosynaptic depression was simultaneously induced at lateral path synapses (20 min fEPSP: −35 ± 5%, p<0.01; 48 h fEPSP: −24 ± 4%, p<0.01, n = 8; Fig. 2A) by the stimulation paradigm.

Tubulin expression in the dentate gyrus molecular layers was compared between control and LTP-stimulated hemispheres (Fig. 2B,C). Similar to the naïve rats, a gradient in the expression of tubulin, decreasing from the IML to the OML, was observed in both the control and LTP hemispheres (p<0.01). There was no significant main effect of LTP induction, or an LTP x layer interaction in the relative distribution (Fig. 2C). Further, there was no difference in tubulin expression between the LTP-stimulated and control hemispheres for any layer (p  =  0.35; Fig. 2D). Thus, in addition to being consistent between hemispheres within each layer in control animals, tubulin expression was not changed by LTP at 48 h after its induction. Accordingly, in our subsequent investigations of glutamate receptor subunit expression after LTP induction, tubulin was used as a loading control for across-hemisphere comparisons (Fig. 3C, 4D, 5C). Tubulin was also used as a loading control in some within-hemisphere comparisons (Fig. 4C), but as a within-hemisphere gradient in tubulin expression was evident, analyses were also carried out without tubulin as a loading control (Fig. 3B, 4B, 5B).

### GluN1 expression is increased in the MML after LTP

We have shown previously that the NMDAR subunit, GluN1, is elevated in whole dentate gyrus homogenates and synaptoneurosomes 48 h after LTP [29]. To determine whether this increase is localised to the LTP-stimulated dendritic zone of the dentate gyrus, GluN1 expression was also investigated in the microdissected molecular layer subregions (Fig. 3A–C). No significant change in the expression of GluN1 in either MML or OML, relative to the IML, was observed (Fig. 3B). However, there was a small but significant increase in the MML expression when compared to the control hemisphere in tubulin-normalised samples (MML: 15 ± 4%, p<0.01, n = 8; Fig. 3C). No differences in expression were detected between the LTP- and control-hemisphere IMLs or OMLs (Fig. 3C). These data indicate that the LTP-associated increase in GluN1 expression that we have observed previously at 48 h post-induction [29] occurs specifically in the stimulated region of the dendritic arbor.

### GluA1 protein is increased in the MML 48 h after LTP induction

The levels of the AMPAR subunits GluA1 and GluA2 were investigated in the IML, MML and OML 48 h after LTP induction. As IML synapses do not display heterosynaptic effects from medial perforant path stimulation at this time point using our protocol [30], expression of each protein was analysed relative to the levels in the ipsilateral IML as an internal control. Our analysis showed that GluA1 expression in the MML was significantly elevated in the tetanised hemisphere (153 ± 15% of IML, n = 7) relative to the control hemisphere MML (112 ± 11% of IML, p<0.01, n = 7) 48 h post-LTP induction, but there was no effect of LTP on GluA1 levels in the OML (p = 0.67, n = 7; Fig. 4A,B). To confirm the elevation in GluA1 protein in the MML, expression data were also analysed using tubulin as a loading control prior to normalisation to the ipsilateral IML. This analysis showed greater GluA1 expression in the tetanised hemisphere MML (199 ± 34% of IML, n = 7) relative to the control hemisphere MML (145 ± 19% of IML, p<0.05 (single-tailed t-test), n = 7; Fig. 4C). Additionally, we made direct comparisons of tubulin-normalised protein levels between hemispheres for each layer. Here, a sizeable but non-significant increase in tubulin-normalised GluA1 expression was observed (30 ± 21%, p = 0.11, n = 7; Fig. 3D).

To determine whether LTP specifically affected GluA1 levels in the MML or if there was a more general layer specific alteration in AMPA receptor subunits we investigated whether GluA2 levels were likewise affected. While a gradient of GluA2 expression was detected, comparable to the regional tubulin expression pattern (Fig. 5A,B), no significant LTP-related changes were observed in the relative distribution of GluA2 protein (MML, p = 0.25, n = 6; OML, p = 0.880, n = 6; Fig. 5A–B), nor were there any differences detected in direct between-hemisphere comparisons (p>0.4, n = 6; Fig. 5C). These data indicate that the redistribution of GluA1 protein seen in Fig. 4B,C occurred independently of changes in GluA2, and may therefore be representative of an accumulation of GluA1 homomeric AMPARs in the MML.

### LTP-independent distribution of receptor subunit proteins

It is noteworthy that between-layer comparisons revealed differential distributions of GluA2 and GluN1 protein across the molecular layer independent of LTP induction. Expression of GluA2 in the OML was significantly lower than the IML across both LTP and control hemispheres (LTP OML 69 ± 14% of IML, control OML 61 ± 13% of IML, overall difference from IML p<0.05, n = 6), with a similar trend when compared to the MML (LTP MML 114 ± 21% of IML, control MML 100 ± 22% of IML, overall difference from OML; p = 0.06, n = 6; Fig. 5B). As GluA1 subunits were equally distributed across the layers (p>0.23 for OML compared to IML and MML), there may be fewer GluA2/GluA3 type AMPARs in the distal zone of the molecular layer, as ∼90% of AMPARs in the hippocampus consist either of GluA1/GluA2 heteromers or GluA2/GluA3 heteromers [31]. It was not possible to confirm this implication through analysis of GluA3 level as we were unable to detect GluA3 protein in LMD tissue using the commercially available antibodies tested.

GluN1 expression was significantly lower in the OML when compared to both the IML and the MML, irrespective of LTP induction (p<0.01, n = 7; Fig. 3B). As the GluN1 subunit is a core requirement of functional NMDARs [32], there may be fewer NMDARs at synapses in the distal zone of the molecular layer.

## Discussion

LMD was pioneered in the 1990s at the United States National Cancer Institute for the purpose of separating cancerous cells from normal cells in heterogeneous carcinomas [33], [34], [35] and is typically used as a diagnostic tool for nucleic acid studies, as the minute amounts of nucleic acid isolated from individual or small numbers of cells can be routinely amplified using the polymerase chain reaction. Due to the fact that it is currently not possible to amplify proteins in a similar fashion, it is a much more onerous task to analyse protein levels in cells and tissues collected by LMD because of the large amount of material required. Of the 2000+ publications utilising laser-based tissue dissection, not more than 10% have been carried out using LMD to investigate protein expression [23], [36]; in the rat dentate gyrus, only two protein-based studies has been carried out previously: one investigating actin levels in the middle molecular layer [24] and one investigating caspase enzyme expression in granule cells [25]. Here we have shown that lower abundance synaptic proteins, namely GluA1, GluA2, GluN1 and PSD-95, as well as the cytoskeletal element tubulin, can be detected in dentate gyrus microdissected tissue using Western blot analysis.

Using LMD we were able to investigate the expression of glutamate receptor proteins in the activated zone of the dentate gyrus dendritic arborization 48 h following LTP-inducing stimulation of the medial perforant path, giving enhanced anatomical resolution to our previous investigations that employed fractionations made from the whole dentate gyrus [14]. Using LMD we found that GluA1 expression was elevated in the LTP-expressing MML relative to the level in the IML, which prior evidence indicates does not display persistent plasticity at this time point in response to the protocols used [30]. Similarly, GluN1 expression was increased in the MML of LTP-hemispheres compared to the MML of control hemispheres. These data are consistent with our previous observations that GluN1 and GluA1 levels are increased in homogenates from the whole dentate gyrus at this time-point [14], [29]. The present data indicate that not only does LTP induction lead to a long-lasting increase in expression of glutamate receptor proteins, but that these proteins accumulate specifically in the region of stimulated synapses. The input-specific property of LTP that contributes to its appeal as an information storage mechanism must also be a property of its expression and maintenance mechanisms. Indeed, it has recently been demonstrated that following contextual fear conditioning in transgenic mice expressing a GluA1-GFP construct under the control of the immediate-early gene *c-fos* promoter, GluA1-GFP was recruited specifically to hippocampal CA1 neuron mushroom spines [37] which have synapses thought to be mature, stable and information-storing [38].

Specific delivery of GluA1 and GluN1 proteins to the LTP-expressing region of the molecular layer can be achieved through proposed synaptic tagging and capture mechanisms [39], [40], [41], [42], in which a molecular ‘tag’ generated at activated synapses causes the synaptic capture of plasticity-related proteins that are synthesized centrally or by dendritic protein synthesis machinery. While we have shown here that receptor proteins are targeted to that region of the dendritic arborisation containing potentiated synapses, it is not possible from our data to determine if these receptors are being delivered to postsynaptic membrane, as our microdissected tissue samples contain dendritic neuropil rather than sub-fractionated synapses only. The limited amount of tissue obtained from laser microdissection precludes the subcellular fractionation required to determine the synaptic versus extrasynaptic versus intracellular localisation of the accumulated receptors observed in this study.

Interestingly, no increase in GluA2 was observed in the MML. This is consistent with our previous finding that GluA2 is unchanged in dentate gyrus synaptoneurosomes and PSD fractions prepared 48 h after LTP induction [14]. Taken together with our contrasting observation that GluA2 is elevated in whole dentate homogenates at this time point[10], these data suggest that any GluA2 increase is confined to the cell bodies of either granule cells or hilar neurons. Thus, the present data appear to indicate that elevated GluA1 levels in the MML represent Ca^2+^-permeable GluA1 homomeric receptors. Hitherto, it has been shown that GluA1 homomers undergo rapid and transient membrane insertion following LTP, before being replaced by GluA1/GluA2 [43] or GluA2/GluA3 heteromers [7]. While GluA3 expression in microdissected subregions was not determined here, as the antibodies used did not reliably detect GluA3 protein, it is unlikely that GluA2/GluA3 receptor levels are elevated, as no increase was detected for GluA2. This indicates that if GluA2/GluA3 receptors do indeed replace GluA1/GluA2 types following LTP in the dentate gyrus, these receptors would need to be drawn from the pool already present in the vicinity of potentiated synapses. Thus, it may be that the increase in GluA1 reflects accumulation of GluA1-homomeric AMPA receptors in nonsynaptic regions, from which they may be recruited for further bouts of LTP [43], [44], [45].

A distinct gradient in both GluN1 and GluA2 expression was observed in the molecular layer, decreasing from the inner and middle molecular layers toward the outer layer. While NMDAR number is considered an indicator of synaptic density [46], it is unlikely that our data reflect a gradient in the number of synapses onto granule cell dendrites, as a previous stereological study has shown that axospinous synapse density is consistent across the molecular layer of the rat dentate gyrus [47]. Dentate gyrus interneurons receive excitatory perforant path inputs that include NMDAR components [48], [49], so the possibility that there is a gradient in the density of perforant path synapses onto interneuronal dendrites in the molecular layer cannot be discounted.

The gradient for GluA2 in the absence of a gradient for GluA1 may reflect a difference in the number of GluA2/GluA3-containing AMPARs between the different zones, and thereby indicate a gradient in synaptic size across the molecular layer [46]. Alternatively such a difference in receptor number could be due to different reserve receptor pool populations [50], [51], [52], [53]. It is unlikely that glutamatergic synapses onto interneurons contribute to the GluA2 expression gradient, as GluA2 mRNA is only expressed at low levels by interneurons and GluA2 immunoreactivity is undetectable [54].

LTP induction in the rat dentate gyrus *in vivo* requires NMDAR activation [55], and is the key event in the initiation of LTP for both medial and lateral perforant path synapses [56], [57]. One explanation for the difference in GluN1 expression across the molecular layer is a difference in NMDAR density at synapses in the different zones. A consequence of such a difference could be associated with differences in plasticity induction thresholds between medial and lateral perforant path synapses. Although asymmetry in the nature of plasticity between the medial and lateral portions of the perforant path has been reported [17], further electrophysiological study into potential asymmetries in plasticity thresholds are warranted.

Pharmacological studies have suggested that the induction of LTP may be specifically dependent on GluN2A-containing NMDARs, and LTD on GluN2B-containing receptors [58], [59], [60], [61], although these conclusions have been challenged by evidence that GluN2B-type receptors can induce LTP [62], [63], [64], [65]. Recent work has indicated that GluN2A-type activation is required for LTP induction while the presence of GluN2B-type receptors is required for the synaptic recruitment of plasticity-relevant molecules [66]. Future LMD investigations should attempt to ascertain whether there is asymmetry in the distribution of GluN2A versus GluN2B NMDAR types across the dentate gyrus molecular layer, and whether this correlates with any asymmetries in plasticity induction thresholds.
